# The Nerve Centers and the Teeth

**Published:** 1885-11

**Authors:** J. Smith Dodge

**Affiliations:** New York.


ARTICLE II.

### THE NERVE CENTERS AND THE TEETH.

BY J. SMITH DODGE, JR., M. D., D. D. S., NEW YORK.

The dentist need not look beyond his own field to find ample proof of nerve influence on the teeth. As regards the influence of maternal conditions during gestation on the tooth-germs of the fetus, the evidence is beyond doubt. The journals have for years contained reports in which successive children of the same mother showed different qualities of teeth which corresponded with difference of maternal regimen. Most commonly the difference is spoken of as a varying supply of lime, the use of unbolted flour, the administration of lime-salts, removal to a lime-stone region. But these are likely also to be causes of improved general health, and so of increasing neural energy; and this view is confirmed by other testimonies which give the same results from other methods of general improvements. The members of the Odontological Society will remember Dr. Rich's account of the benefit exhibited in the teeth of children from gymnastic training of their mothers. So it looks to me plain that we are not to regard this matter as one of more or less material for tooth-building, but as concerning the ability of enamel organs and odonto blasts to work up the material at hand into perfect tissue.

But the influence of maternal organism becomes less immediate at birth, long before the permanent teeth have completed their structure; and at this we must look to the nerve-centers of the child. Now, I can hardly conceive any observing dentist doubts the influence of infantile sickness or health on the character of the permanent teeth. The various forms of atrophied enamel are now, as they have long been, considered the results of general disease during their formation; and the different qualities of dentine and of enamel, without defect of form, which so often distinguishes teeth of simultaneous development from those formed easlier or later in the same mouth, can frequently be directed traced to grave fluctuations of general health. Indeed, I am convinced that the inferiority of the first permanent molars is largely due to the general disturbances which so commonly accompany the first dentition.

A step further brings us to consider the teeth that belong to the so-called nervous temperament. When this phrase means, as it did in the old physiology, a harmonious organism thoroughly ruled by a well-balanced nervous system, we find admirable teeth—small, a little spaced, of a dark but translucent yellow, and very enduring. But when the phrase means, as it does in popular use, a fragile organism. overridden and devoured by hypertrophied nerve-centers, we find, together with the vivacious spirit, the brilliant eye, the transparent skin, and the weak lungs, pearly teeth, which you can almost see through, beautiful while they last, but easy to cut and doomed to decay.

Thus far we have been concerned with the original formation of the teeth. But turning now to their subsequent history, we shall find numerous illustrations of our theme. We have all groaned in spirit over the teeth of children hard driven in study and pleasure. The original sensibility rises to the point of a positive neurosis, and month by month they melt like snow. We all know the destructive result to the mother's teeth during pregnancy and of the depression of vital energy which so often follows it. Something has evidently enfeebled the teeth. In New York the fashionable world dissipates all winter, and aims to recuperate in summer. I have found it a common thing that the same woman's teeth, which were fairly normal in the fall, become sensitive and decaying by spring; and I have often found the condition reversed again after a summer reasonably spent. Finally, in old age, teeth which have withstood the work of a life-time frequently succumb to rapid diffuse, painless decay, which forces on you the conviction that somehow life has half deserted these outworks and abandoned them to the enemy.*

Now, all these instances bear on the same point, namely, that whatever at any period of life exhausts or prevents the action of the great nerve-centers impairs the power of the teeth to resist the ever-present causes of decay. More might be added, but it is needless. I am convinced the nerve-centers hold sovereign sway over the formation of

*We think this is not frequent. As a rule, the teeth of the aged is not as subject to caries as the teeth of the young.—ED. ITEMS.

the tooth and the nutrition of its tissues; so that without proper and constant influence from those centers, the formative elements are unable to perfect their work, and if this sustaining power be impaired or withdrawn the teeth become an easy prey to the manifold agencies of ruin which surround them.

If these views be correct, we are confronting, in the ruinous decay of teeth, only one angle of a many sided subject; and indeed we can hardly stop short of philosophizing on the entire structure of modern society. Formerly man ruled the world by muscle and bone. The strong arm, the brawny back, the impetuosity of animal vigor made the ruling men and the ruling races. Now man rules the world by his nervous system. Clearness of understanding, tenacity of purpose, energy of will, make now the kings of men. It comes to pass, therefore, that those races rule which can put forth most forcibly and sustain longest the powers of the nerve-centers. Each man, each race, rivals the others, till we begin in our time to see a one-sided development of the best races, which is the exact reverse of what prevailed a thousand years ago, when the leaders of the world were brutal giants, ruling by bone and muscle, with intellect and sentiment shockingly atrophied. To-day the other end is up. It is brain and will that rule, dwelling perhaps in a hundred and twenty pounds of anguished flesh, tormented with dyspepsia and insomnia. That this modern man has bad teeth, and his children worse, is only a part of the same physical atrophy, the price he pays for hypertrophied nerve-centers.

I have spoken of the *man* of to-day,but the same thing is true of our women. Having within the last few generations come, into the sovereignty of the social world, how infinitely more delightful they make it by their charms of mind and sentiment than their predecessors ever did by mere force of physical charm. That is to say, they exactly parallel the man. They have replaced the former reign of beautiful flesh by a nobler reign of delicate and exalted nerve-centers, which, as before, cultivation and rivalry push to the last extreme.

Now, when this man and this woman combine their energies for the initiation of a new life, it may easily be foreseen that their nervous systems, racked and exhausted by these perpetual struggles, will have too little latent energy remaining to endow their offspring. You cannot eat your cake and have your cake. Neither can you spend your neural force in the service of individual life and at the same time respond normally to the large demands of reproduction. It is an old observation that all unusal expenditures of brain-force tend to produce sterility. For the same reason they lower the quality of the children whose conception they do not prevent. And this deterioration, of course, follows the lead of the parental organism. Everything else is stinted (since there is not enough for all) that the nerve-centers may be largely and rapidly developed; and in the first rank of the organs which suffer is the entire dermal system, which includes the teeth. The skin itself, the hair, the nails, as well as the teeth, becomes more delicate, incapable of resistance. And presently comes the second stage of this sad, eventful history. This one-sided little creature is hardly aware of his new surroundings before parents, nurses, and friends begin to stimulate his over-grown nerve-centers, delighting in every evidence of precocity, and pushing him step by step through successive studies and pleasures far beyond his years. What city dentist has not been dismayed to hear of the school and home studies, music lessons, dancing lessons, matinees, balls, and all the rest, which make it difficult to find time for the care of this little darling's half-constructed teeth. And who has not read the end of it all in the mother's elegant mourning? The painful, perishing teeth are an integral part and a fair index of the entire physical condition.

And so we come round, through all these considerations, to perceive that the increasing frailty of the teeth is not due to this or that matter of diet or regimen, but is part of the general fact that parents and children alike spend so much nerve-force on the world without that they have not enough left to nourish rightly the tissues within.

But it is time to ask if there be any remedy. Thanks to the marvelous recuperative power of our race, there is. I believe it may be possible, even in the individual,and certainly in the second generation, to turn inward this stream too prodigally wasted without, and utilize for self-support the vast neural power which modern times have developed. Where the income is assured it is only necessary to restrain waste, and wealth will follow. The last twenty years have made a promising beginning in the management of children in dress, diet, ventilation and exercise. But these are only the fringes of the matter. Two things remain of prime importance: First, let conception be to every wife the signal of a new, an imperative, and an honorable duty, namely, the duty to cultivate all her energy of mind and body in quiet, simple ways, and to stop all waste of nervous force, whether in pleasure, work, or worry—that the new life which feeds on her may satisfy its great requirements from an ample store. Secondly, let precocity be considered a danger to the child and somewhat a disgrace to the parent—a thing not always avoidable, but never to be desired or forwarded.

How far off may be the realization of such dreams, it would be foolish to predict. But as one who has an abiding faith in the future, I am glad to observe the beginnings of better things, and I do not hesitate to prophesy that some day all this and more will come to pass. For the present the disproportionate strain put on the nerve-centers wears out the organism that should serve all the purposes of life. The tool crumbles under the stroke. But let a few generations of proper regimen bring up the general development to match this enormous energy, and the world will see a race of men whose thought and sinew will tame the elements, and whose teeth will grind for a hundred years the food that feeds them.—Trans. Odon. So., N. Y., in *Cosmos*.

